# In this month’s *Bulletin*

**DOI:** 10.2471/BLT.15.000715

**Published:** 2015-07-01

In the editorial section, Hildy Fong & Eva Harris (438) encourage the fair distribution of health technology. Arthur Chioro et al. (439) discuss future action for managing antimicrobial resistance.

In the news section, Gary Humphreys and Fiona Fleck report on the use of the media in public health education (442–443). Fiona Fleck interviews Pali Lehohla on his involvement in the upcoming post-2015 development goals (444–445).

## Africa

### Training rabies-control officers

Hervé Bourhy et al. (503–506) orchestrate a comprehensive disease control training programme.

## Kenya, Malawi, South Africa, Uganda, the United Republic of Tanzania and Zimbabwe.

### Which policies work?

Kathryn Church et al. (457–467) compare national HIV policies in six countries.

## Chile

### School bans on smoking

Xiaolin Wei et al. (407–416) measure patients’ perceptions of primary care in Shanghai and Shenzhen.

## United Republic of Tanzania

### Improving maternal and newborn health in rural areas

Andrea B Feigl et al. (468–475) evaluate the effectiveness of a high-school ban on smoking.

## Haiti

### Tuberculosis following a natural catastrophe

Serena P Koenig et al. (498–502) investigate a rise in tuberculosis cases following the 2010 earthquake.

## India 

### Getting value for money in hospitals

Susmita Chatterjee & Richard A Gosselin (476–482) compare surgical interventions using two disability weight databases.

## Global

### Improving air quality data 

Kendra N Williams et al. (507–508) call for new questions in household surveys on cooking and fuel collection.

### Success in shared toilets 

Thilde Rheinländer et al. (509–510) argue for the inclusion of shared facilities in development goal measures.

### Tackling drug-resistant tuberculosis

Helen S Cox et al. (491–497) describe the need for new treatment regimens.

### Tracking schistosomiasis in water buffalo 

Jose Ma M Angeles et al. (511–512) outline the need for surveillance of the animal reservoir.

### What is wealth? 

Daniel J Hruschka et al. (483–490) test a new metric for global wealth estimates.

### What’s making people fat? 

Stephanie Vandevijvere et al.****(446–456) investigate the impact****of increased food supply on****global obesity.

